# Inhibition Mir-92a Alleviates Oxidative Stress and Apoptosis of Alveolar Epithelial Cells Induced by Lipopolysaccharide Exposure through TLR2/AP-1 Pathway

**DOI:** 10.1155/2020/9673284

**Published:** 2020-09-16

**Authors:** Jian Cui, Huanhuan Ding, Yongyuan Yao, Wei Liu

**Affiliations:** ^1^Department of Intensive Care Unit (ICU), People's Hospital of Rizhao, Shandong Province, China; ^2^Department of Cardiology, People's Hospital of Rizhao, Shandong Province, China

## Abstract

**Objective:**

To probe into the role of miR-92a in alleviating oxidative stress and apoptosis of alveolar epithelial cell (AEC) injury induced by lipopolysaccharide (LPS) exposure through the Toll-like receptor (TLR) 2/activator protein-1 (AP-1) pathway.

**Methods:**

Acute lung injury (ALI) rat model and ALI alveolar epithelial cell model were constructed to inhibit the expression of miR-92a/TLR2/AP-1 in rat and alveolar epithelial cells (AECs), to detect the changes of oxidative stress, inflammatory response, and cell apoptosis in rat lung tissues and AECs, and to measure the changes of wet-dry weight (W/D) ratio in rat lung tissues.

**Results:**

Both inhibition of miR-92a expression and knockout of TLR2 and AP-1 gene could reduce LPS-induced rat ALI, alleviate pulmonary edema, inhibit oxidative stress and inflammatory response, and reduce apoptosis of lung tissue cells. In addition, the TLR2 and AP-1 levels in the lung tissues of ALI rats were noticed to be suppressed when inhibiting the expression of miR-92a, and the AP-1 level was also decreased after the knockout of TLR2 gene. Further, we verified this relationship in AECs and found that inhibition of miR-92a/TLR2/AP-1 also alleviated LPS-induced AEC injury, reduced cell apoptosis, and inhibited oxidative stress and inflammatory response. What is more, like that in rat lung tissue, the phenomenon also existed in AECs, that is, when the expression of miR-92a was inhibited, the expression of TLR2 and AP-1 was inhibited, and silencing TLR2 can reduce the expression level of AP-1.

**Conclusion:**

MiR-92a/TLR2/AP-1 is highly expressed in ALI, and its inhibition can improve oxidative stress and inflammatory response and reduce apoptosis of AECs.

## 1. Introduction

Acute lung injury (ALI) is an acute respiratory distress syndrome (ARDS) with clinical characteristics of acute hypoxic respiratory failure and bilateral pulmonary infiltration caused by multiple factors in and out of the lung. Due to the lack of specific treatment, the mortality rate can reach up to 40% [1–3]. Alveolar epithelial cells (AECs), as the main sites for gas exchange, are also one of the main components of respiratory barrier [4]. During ALI, AECs are always found damaged more, the generation of surfactant reduced, lung compliance decreased, and gas exchange blocked [5, 6].

Short noncoding RNA (miRNAs) are a class of small RNA with a length of about 18-25 nucleotides. By binding to messenger RNA (3′-UTR) and inducing RNA silencing or degradation through miRNA-induced silencing complexes, they negatively regulate protein-coding genes' expression and participate in the regulation of various cellular processes, including inflammatory responses [7, 8]. In recent years, multiple literature has reported that miRNAs have a part to play in ALI as markers of acute lung injury and diffuse alveolar injury [9]. A case is that in the study of Song et al. [10], miR-34a could target FoxO3 to inhibit autophagy of alveolar type II epithelial cells in ALI and reduce the damage of lipopolysaccharides (LPS)-induced ALI. MiR-92a has also been found to act on development of inflammatory responses in ALI rats, and its inhibition can reduce the secretion of proinflammatory factors and improve inflammatory responses [11]. MiRNAs are important regulators of Toll-like receptor (TLR) signaling, among which TLR2 has been reported as one of the targets of miR-92a, which can alleviate liver fibrosis caused by Schistosoma japonicum [12]. In addition, Lai et al. [13] demonstrated that TLR-mediated decrease in miR-92a expression can promote the production of inflammatory cytokines in TLR-induced macrophages. What is more, Fei et al. [14] revealed that in ALI, glycyrrhizic acid can block the TLR-2 signal cascade to inhibit the inflammatory response induced by ischemia-reperfusion lung injury. Activator Protein-1 (AP-1) is an upstream regulator of interleukin-4 (IL-4), which also participates in the ALI process. Khan et al. [15] showed that Anomalin could inhibit AP-1 to relieve mechanical pain and inhibit leukocyte infiltration in ALI rats. And AP-1 is regulated by TLR2 [16, 17].

Based on preceding research, we hypothesized whether there is a signaling axis such as miR-92a/TLR2/AP-1 that is effective in the occurrence and progression of ALI. Hence, in this study, we analyzed the effect of miR-92a/TLR2/AP-1 on ALI AECs.

## 2. Materials and Methods

### 2.1. Rat Source

A total of 100 healthy Sprague-Dawley rats, aged 10 weeks and weighed 250-300 g, were obtained from the Experimental Animal Center of Harbin Medical University. The rats were kept at room temperature of 20-25°C, relative humidity of 40%-70%, normal 12 h circadian rhythm, and they were free to eat and drink. All animal experiments in present study were approved by the Animal Care and Use Committee of our hospital and followed the guidelines of the Council for International Organizations of Medical Sciences (CIOMS).

### 2.2. ALI Rat Model Construction and Observation Index

All the rats were grouped into a control group (CG), a model group (MG), a miR-92a inhibitor group, a TLR2(-) group, and an AP-1(-) group at random, with 20 in each group. TLR2 and AP-1 gene knockout were performed on the rats of TLR2(-) group and AP-1 group by Sigma company, while the CG was left untreated. The MG, miR-92a inhibitor group, TLR2(-) group, and AP-1(-) group were intratracheally infused with 5 mg/kg LPS. Meanwhile, miR-92a inhibitor group was injected via tail vein with a concentration of 100 mg/kg pretreated with liposome 2000 miR-92a inhibitor carrier, and miR-92a inhibitor vector was designed and synthesized by Sigma Company. Twenty-four hours later, pentobarbital sodium (45 mg/kg, Sigma) was intraperitoneally injected into the rats to anaesthetize and kill according to the guidelines for the Care and Use of Laboratory Animals, and the lung tissues were isolated. Then, the W/D ratio, apoptosis rate, oxidative stress, and inflammatory factors in the lung tissues of rats in the five groups were detected.

### 2.3. Detection Methods

W/D Ratio. After removing both lungs from the bronchi of free rats, the left lung was taken as the object of examination. The fresh lung tissue was weighed and baked to constant weight in an oven at 80°C. The W/D ratio = dry weight/wet weight.

Apoptosis Rate. Half of the right lung tissue was cut into pieces and centrifuged at 300 × g for 1 min at 4°C in a filter centrifuge tube with a diameter of 35 *μ*m. After removing the supernatant, phosphate buffer solution was added to resuspend the precipitated cells, and the cell concentration was adjusted to 1 × 10^6^ ml. Then, AnnexinV-FITC and PI were added successively to incubate in the dark at room temperature for 5 min, followed by the detection conducted by FACSCalibur flow cytometry (BD Biosciences, CA, USA). The experiment was repeated 3 times to take the average. The Annexin V-FITC/PI apoptosis detection kit was purchased from Invitrogen, USA, under the article number V35113.

Detection of Oxidative Stress and Inflammatory Response Levels. The remaining half of the right lung tissue was placed in a glass homogenate tube, and the tissue was ground into a pulp. The levels of tumor necrosis factor *α* (TNF-*α*), interleukin-6 (IL-6), interleukin-10 (IL-10), malondialdehyde (MDA), and superoxide dismutase (SOD) in lung tissues were detected by ELISA with reference to the kit instructions. The MDA, TNF-*α*, IL-6, and IL-10 ELISA kits were purchased from Guduo Biotechnology Co., Ltd., Shanghai, China, with the article numbers of GD-BN1921, GD-DS1716, GD-DS1726, and GD-DS1731, respectively, and SOD ELISA kit was obtained from Jingkang Bioengineering Co., Ltd., Shanghai, China, with the article number of JLC2390.

### 2.4. Cell Experiment

AECs A549, numbered ATCC ®CCL-185 cells, were acquired from American Type Culture Collection (ATCC). The cells were culture in the medium consisting of FMUI 12K medium (ATCC, 30-2004) +10% fetal bovine serum (ATCC, 30-2020) under 95% air and 5% carbon dioxide (CO2), at a temperature of 37°C.

### 2.5. Cell Grouping and Treatment

Cells were divided into 5 groups, namely the blank group (BG), LPS group, inhibitor group, si-TLR2 group, and si-AP-1 group. Concerning the treatment of cells in each group, those in the BG were left untreated, while those in the inhibitor group were transfected with miR-92a inhibitor vector, those in the si-TLR2 group were transfected with si-TLR2 vector, and those in the si-AP-1 group were transfected with si-AP-1 vector. The si-TLR2 and si-AP-1 vectors were designed and synthesized by Sigma, and the corresponding vectors were transfected by means of Lipofectamine 2000 kit (Thermo Fisher China). In addition to the BG, the other 4 groups of cells were supplemented with 1 g/ml of LPS. After 24 hours of culture, the apoptosis rate and changes in oxidative stress indicators in the cells were detected, with the same detection method as above.

### 2.6. qRT-PCR

Trizol kit (Invitrogen) was applied to separate the Total RNA from tissues/cells. The EasyScript One-Step RT-PCR SuperMix kit was acquired from Transgen Biotechnology, Co., Ltd, Beijing, China, and the specific detection steps were referred to the kit instructions. RNA Template: 1 *μ*g, Forward GSP (10 *μ*M): 0.4 *μ*L, Reverse GSP (10 *μ*M): 0.4 *μ*L, 2∗One-Step Reaction Mix: 10 *μ*L, EasyScript One-Step Enzyme Mix: 0.4 *μ*L, and RNase-free Water was added to complete the reaction volume of 20 *μ*L. The reaction conditions were 40°C for 30 min, 94°C for 5 min, 94°C for 30s, 60°C for 30s, 72°C for 2 kb/min, and 72°C for 10 min, totaling 40 cycles. Three replicate wells were set up in the experiment, and U6 was used as the internal reference for the reaction. The results were analyzed by 2^-*△*Ct^ method.

### 2.7. Western Blot

The protein in the cells/tissues was extracted by repeated freeze-thaw method, and the protein concentration was determined with the aid of BCA. Next, the protein was made to 4*μ*g/*μ*L, electrophoretically separated by 12% SDS-PAGE before it was processed under the initial voltage of 90 V, and then an increased voltage of 120 V to shift the sample to the suitable site on the separation gel. Upon the completion of electrophoresis, the membrane was transferred, with 100 V constant pressure for 100 min and sealed for 60 min at 37°C. Then, the membrane was placed in 5% skim milk for sealing before immune reaction. The membrane was subsequently cultivated overnight at 4°C after adding with primary antibody (1 : 1000), followed by warm washing with PBS three times the next day, 5 min each, and then incubated with secondary antibody (1 : 1000) at room temperature for 1 hour. After that, ECL luminescent reagent was developed and fixed. Quantity One software was employed for statistical analysis of the bands after film scanning, and the protein's relative expression level was equal to the gray value of the bands/the grays value of the internal parameters. BCA protein kit, trypsin, and ECL luminescence kit were all acquired from Thermo Scientific™, with the corresponding article number of 23250, 35055, and 90058. Rabbit anti-TLR2 monoclonal antibody, rabbit anti-AP-1 polyclonal antibody, rabbit anti-bax, goat anti-rabbit IgG secondary antibody, and bcl-2 monoclonal antibody were obtained from Abcam, USA, under the article numbers of ab209217, ab21981, ab32503, ab185002, and ab6721, respectively.

### 2.8. Statistical Analysis

SPSS19.0, which was purchase from Chicago, IL, USA, was employed for statistical analysis of the collected data. The measurement data was described in the form of mean ± SD. The comparison between two groups was conducted using Student's *t*-test, while that among multiple groups was carried out by one-way ANOVA. LSD test was adopted for post hoc test. Two-tailed *P* < 0.05 was considered statistically significant. Graph-Pad Prism 8.0 (La Jolla, CA) was responsible for picture drawing.

## 3. Results

### 3.1. Effects of miR-92a/TLR2/AP-1 on ALI Rats

#### 3.1.1. Analysis of miR-92a/TLR2/AP-1 Level in 5 Groups of Rats

**Figure 1:**
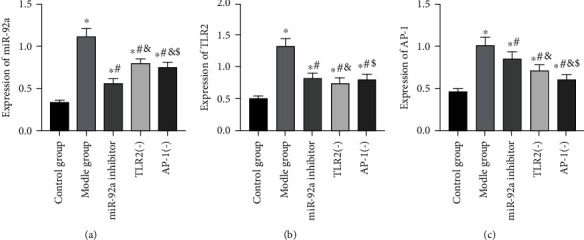
Analysis of miR-92a/TLR2/AP-1 level in 5 groups of rats. (a) Changes in miR-92a expression level in rat lung tissues. (b) Changes in TLR2 expression level in rat lung tissues. (c) Changes in AP-1 expression level in rat lung tissues. ∗ indicates *P* < 0.05 compared with the CG, # indicates *P* < 0.05 compared with the MG, & indicates *P* < 0.05 compared with the miR-92a inhibitor group, and $ indicates *P* < 0.05 compared with the TLR2(-) group.

After LPS stimulation, the level of miR-92a/TLR2/AP-1 in the lung tissues in the MG was noticeably higher than that in the CG (*P* < 0.05). When compared with the MG, the miR-92a in the lung tissue in the miR-92a inhibitor group was decreased (*P* < 0.05), the TLR2 in the TLR2(-) group was declined (P<0.05), and the AP-1 in the AP-1(-) group was reduced (*P* < 0.05). Moreover, it was found that after inhibiting the expression of miR-92a, the expression levels of TLR2 and AP-1 in the lung tissues of the miR-92a inhibitor group were also lower than those of the MG (*P* < 0.05), and the AP-1 in the TLR2(-) group also went down while inhibiting the expression of TLR2 (*P* < 0.05) (Figure 1).

#### 3.1.2. Changes of W/D Ratio in ALI Rats' Lung Tissues

**Figure 2:**
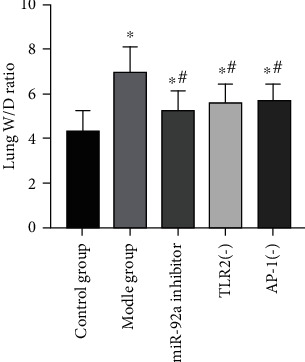
Changes of W/D ratio of lung tissues in ALI rats. ∗ indicates *P* < 0.05 compared with the CG, # indicates *P* < 0.05 compared with the MG, & indicates *P* < 0.05 compared with the miR-92a inhibitor group, and $ indicates *P* < 0.05 compared with the TLR2(-) group.

Compared to the CG, the W/D ratio of lung tissues in the MG increased remarkably after LPS stimulation, but decreased in the miR-92a inhibitor group, TLR2(-) group, and AP-1(-) group after inhibiting the expression of miR-92a/TLR2/AP-1. The W/D ratio of lung tissues differed little among miR-92a inhibitor group, TLR2(-) group, and AP-1(-) group (*P* > 0.05) (Figure 2).

#### 3.1.3. Changes of Apoptosis Level in ALI Rats' Lung Tissues

**Figure 3:**
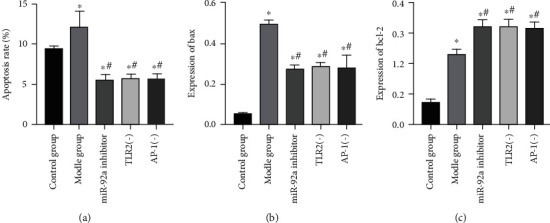
Changes of apoptosis level in lung tissues of ALI rats. (a) Results of apoptosis detection in rat lung tissues. (b) Changes in bax expression level in rat lung tissues. (c) Changes in bcl-2 expression level in rat lung tissues. ∗ indicates *P* < 0.05 compared with the CG, and # indicates *P* < 0.05 compared with the MG.

Compared with the CG, LPS stimulation markedly elevated the apoptosis rate and bax and bcl-2 levels in the MG (*P* < 0.05), while inhibition of miR-92a/TLR2/AP-1 expression resulted in decreased apoptosis rate and bax levels, and increased bcl-2 levels in the miR-92a inhibitor group, TLR2(-) group, and AP-1(-) group than the MG (*P* < 0.05). The level of apoptosis in the lung tissues did not identify any significant difference among the miR-92a inhibitor group, TLR2(-) group, and AP-1(-) group (*P* > 0.05) (Figure 3).

#### 3.1.4. Changes of Oxidative Stress Levels in ALI Rats' Lung Tissues

**Figure 4:**
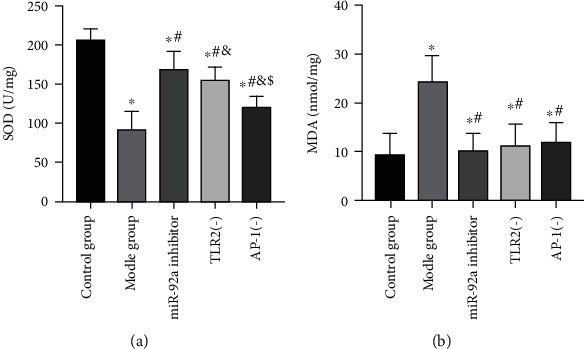
Analysis of oxidative stress levels in lung tissues of rats in 5 groups. (a) Changes in SOD expression level in rat lung tissues. (b) Changes in MDA expression level in rat lung tissues. ∗ indicates *P* < 0.05 compared with the CG, # indicates *P* < 0.05 compared with the MG, & indicates *P* < 0.05 compared with the miR-92a inhibitor group, and $ indicates *P* < 0.05 compared with the TLR2(-) group.

For the purpose of evaluating the effect of miR-92a/TLR2/AP-1 on oxidative stress levels in ALI rats' lung tissues, we observed the changes of oxidative stress in the lung tissues of ALI rats. It turned out that after LPS stimulation, the oxidative stress levels in the lung tissues of rats in the MG rose dramatically, SOD level dropped (*P* < 0.05), and MDA level boosted (*P* < 0.05). However, after inhibiting the expression of miR-92a/TLR2/AP-1, the levels of oxidative stress in ALI rats' lung tissues decreased, the SOD level elevated (*P* < 0.05), and the MDA level declined (*P* < 0.05). In particular, the inhibitory effect of miR-92a on oxidative stress was the most obvious. Among the three groups of miR-92a inhibitor group, TLR2(-) group and AP-1(-) group, the level of SOD in the lung tissues of miR-92a inhibitor group was the highest (*P* < 0.05) (Figure 4).

#### 3.1.5. Changes of Inflammatory Response Levels in ALI Rats' Lung Tissues

**Figure 5:**
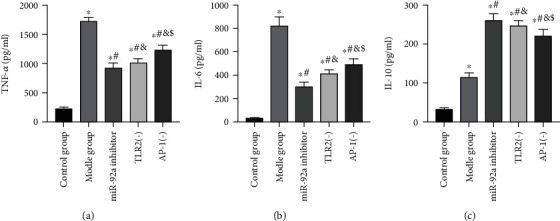
Analysis of inflammatory reaction levels in lung tissues of rats in 5 groups. (a) Changes in TNF-*α* expression level in rat lung tissues. (b) Changes in IL-6 expression level in rat lung tissues. (c) Changes in IL-10 expression level in rat lung tissues. ∗ indicates *P* < 0.05 compared with the CG, # indicates *P* < 0.05 compared with the MG, & indicates *P* < 0.05 compared with the miR-92a inhibitor group, and $ indicates *P* < 0.05 compared with the TLR2(-) group.

Further, we assessed the effect of miR-92a/TLR2/AP-1 on inflammatory response levels in ALI rats' lung tissues. The levels of TNF-*α*, IL-6, and IL-10 in the lung tissues of the MG were obviously increased (*P* < 0.05). Inhibition of miR-92a/TLR2/AP-1 brought lowered TNF-*α* and IL-6 levels (*P* < 0.05), elevated IL-10 level (*P* < 0.05), and suppressed inflammatory response in ALI rats, and miR-92a showed the strongest anti-inflammatory effect. The TNF-*α* and IL-6 in the lung tissues in the miR-92a inhibitor group were lower than those in the TLR2(-) group and the AP-1(-) group, with a higher IL-10 (*P* < 0.05) (Figure 5).

### 3.2. Effects of miR-92a/TLR2/AP-1 on LPS Exposed AECs

#### 3.2.1. Results of miR-92a/TLR2/AP-1 Transfection in AECs

**Figure 6:**
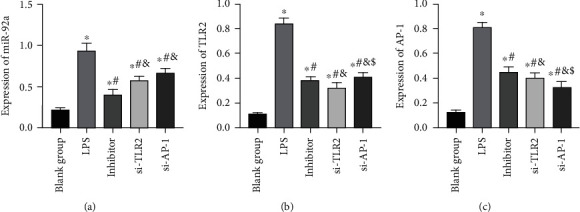
Results of miR-92a/TLR2/AP-1 transfection in AECs. (a) Changes in miR-92a expression level in AECs. (b) Changes in TLR2 expression level in AECs. (c) Changes in AP-1 expression level in AECs. ∗ indicates *P* < 0.05 compared with the BG, # indicates *P* < 0.05 compared with the LPS group, & indicates *P* < 0.05 compared with the inhibitor group, and $ indicates *P* < 0.05 compared with the si-TLR2 group.

After 48 h of transfection, miR-92a, TLR2, and AP-1 levels in cells of inhibitor group, si-TLR2 group, and si-AP-1 group all showed corresponding decreases (*P* < 0.05). In addition, compared with the LPS group, the TLR2 and AP-1 levels in cells of the inhibitor group were remarkably decreased after downregulating miR-92a (*P* < 0.05), while the expression level of AP-1 was also reduced after downregulating the expression of TLR2 (*P* < 0.05) (Figure 6).

### 3.3. Effects of miR-92a/TLR2/AP-1 on Apoptosis Level of LPS Exposed AECs

**Figure 7:**
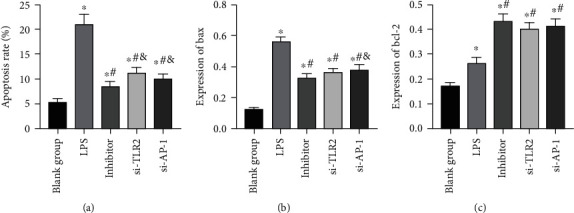
Analysis of apoptosis level of AECs. (a) Apoptosis rate of AECs (b) Changes in bax expression level in AECs. (c) Changes in bcl-2 expression level in AECs. ∗ indicates *P* < 0.05 compared with the BG, # indicates *P* < 0.05 compared with the LPS group, and & indicates *P* < 0.05 compared with the inhibitor group.

After exposure to LPS, the apoptosis level of AECs increased dramatically, and the apoptosis rate and bax and bcl-2 levels in the LPS group were higher than those in the BG (*P* < 0.05). While inhibited miR-92a/TLR2/AP-1 led to decreased apoptosis level of AECs, and the apoptosis rate and bax and bcl-2 levels in the inhibitor group, si-TLR2 group, and si-AP-1 group were lower than those in LPS group, and the bcl-2 level increased (*P* < 0.05) (Figure 7).

### 3.4. Effects of miR-92a/TLR2/AP-1 on Oxidative Stress Levels of LPS Exposed AECs

**Figure 8:**
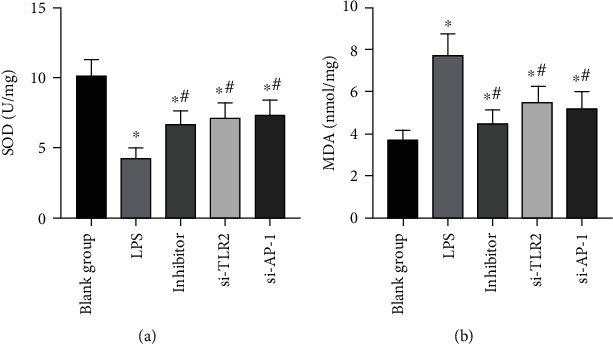
Changes in oxidative stress levels in AECs. (a) Changes in SOD expression level in AECs. (b) Changes in MDA expression level in AECs. ∗ indicates *P* < 0.05 compared with the BG, and # indicates *P* < 0.05 compared with the LPS group.

After LPS stimulation, SOD level in AECs declined (*P* < 0.05), MDA level boosted (*P* < 0.05), and oxidative stress level enhanced. While inhibited miR-92a/TLR2/AP-1 reduced the oxidative stress stimulation of LPS on AECs, increased SOD, and decreased MDA (*P* < 0.05). (Figure 8)

### 3.5. Effects of miR-92a/TLR2/AP-1 on Inflammatory Response Levels of LPS Exposed AECs

**Figure 9:**
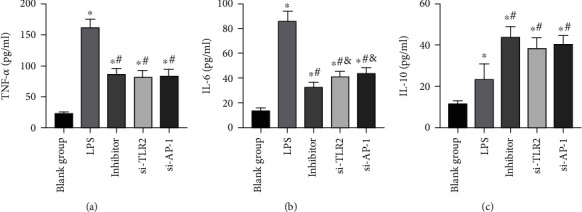
Analysis of inflammatory response levels of AECs. (a) Changes in TNF-*α* expression level in AECs. (b) Changes in IL-6 expression level in AECs. (c) Changes in IL-10 expression level in AECs. ∗ indicates *P* < 0.05 compared with the BG, # indicates *P* < 0.05 compared with the LPS group, and & indicates *P* < 0.05 compared with the inhibitor group.

The level of inflammatory response of AECs in the LPS group was higher than the BG. After being stimulated by LPS, TNF-*α*, IL-6, and IL-10 levels increased noticeably (*P* < 0.05). While after the inhibition of miR-92a/TLR2/AP-1 in cells, the inflammatory response inhibited, TNF-*α* and IL-6 decreased, and IL-10 increased (*P* < 0.05) (Figure 9).

## 4. Discussion

ALI is a refractory respiratory dysfunction disease whose pathogenesis is not yet fully understood, so specific prevention and treatment approaches have been lacking, leading to a high mortality rate globally [18]. MiRNAs exert marked effects on cell development, genomic imprinting, and regulation of cell functions [19]. With the exception of Fu et al. [11], little research in recent years has reported the part miR-92a plays in ALI. The present study once again verified the effect of miR-92a on ALI rats and further analyzed miR-92a's effects on the survival of AECs exposed to LPS. It was found that miR-92a attenuated the oxidative stress response and apoptosis of LPS-induced AECs through TLR2/AP-1 signaling axis.

In this study, we revealed that both inhibition of miR-92a expression and knockout of TLR2 and AP-1 genes could reduce LPS-induced rat ALI, alleviate pulmonary edema, inhibit oxidative stress and inflammatory response, and reduce apoptosis of lung tissue cells. In addition, it was noticed that in ALI rats' lung tissues, TLR2 and AP-1 were suppressed after inhibiting the expression of miR-92a, and the expression of AP-1 was decreased after knocking out the TLR2 gene, suggesting that miR-92a/TLR2/AP-1 might be a signaling axis that exerts marked effects on the occurrence and development of ALI. The regulatory effect of miRNAs on TLR protein has been confirmed in many studies [20], while only Zhao et al. [12] reported that miR-92a could target the regulation of TLR2 expression. Apart from that, it is well established that AP-1 can be regulated by TLR2. As reported by Wan et al. [21], geranyl diphosphate synthase 1 alleviates ventilator-induced lung injury through the TLR2/4/AP-1 signaling axis. Moreover, the TLR2-JNK-AP-1 pathway also plays a crucial part in pulmonary fibrosis caused by Mycobacterium tuberculosis [22]. This also supports our speculation, and we verified this relationship in AECs. We found that inhibition of miR-92a/TLR2/AP-1 also alleviated LPS-induced AEC injury, reduced cell apoptosis, and inhibited oxidative stress and inflammatory response. What is more, like that in rat lung tissues, the phenomenon also existed in AECs, that is, when miR-92a was inhibited, the expression of TLR2 and AP-1 was also inhibited, and silencing TLR2 could reduce the expression level of AP-1. Therefore, it can be found from our study that miR-92a/TLR2/AP-1 is related to the onset and progression of ALI, and inhibition of it can effectively improve ALI symptoms and reduce apoptosis of AECs.

MiR-92a is a gene closely associated with lung disease and has been shown in many studies to be bound up with lung cancer metastasis. For example, Hsu et al. [23] reported in their study that bone marrow-derived cells could release vesicles coated with miR-92a to promote liver metastasis of lung cancer. In the study of Borzi et al. [24], miR-92a was found to promote the proliferation of lung bronchial cells and establish an ecological environment for intralung metastasis of lung cancer. However, earlier evidence [25] only revealed that inhibited miR-92a could promote the WNT1-inducible signaling pathway protein 1 (WISP1) in lung fibroblasts, a fibrogenic medium, but they did not further analyze fibroblast changes, so miR-92a may inhibit the development of pulmonary fibrosis. This suggests that miR-92a has various roles in different lung diseases, but it is unclear whether miR-92a also has a two-way effect in ALI, which needs to be verified by more studies. Although this study studied the role of miR-92a/TLR2/AP-1 in ALI from animal models in vivo and cell models in vitro, further clinical trials are still needed.

To sum up, miR-92a/TLR2/AP-1 is highly expressed in ALI, and its inhibition can improve oxidative stress and inflammatory response and reduce apoptosis of AECs.

## Data Availability

The corresponding data of this manuscript can be available if any researcher required.
